# Reply letter on “Physiological effects of high-flow nasal cannula oxygen therapy after extubation: a randomized crossover study”

**DOI:** 10.1186/s13613-023-01240-8

**Published:** 2024-01-11

**Authors:** Roque Basoalto, Alejandro Bruhn

**Affiliations:** 1 Departamento de Medicina Intensiva, Facultad de Medicina, Pontificia Universidad Católica de Chile, Santiago, Chile; 2 Programa de Medicina Física y Rehabilitación, Red Salud UC-CHRISTUS, Santiago, Chile; 3 CardioREspirAtory Research Laboratory (CREAR), Departamento de Ciencias de La Salud, Pontificia Universidad Católica de Chile, Santiago, Chile; 4 Center of Acute Respiratory Critical Illness (ARCI), Santiago, Chile

We appreciate the interest by Toumi and colleagues in commenting about our recent article [1]. Although we agree with some of their comments and suggestions, we disagree with others.

Toumi et al. indicate that our study lacked a comprehensive approach. They support this criticism in the supposition that patients at high risk of extubation failure were not included, and in the fact that a crossover design was used. First, we did not exclude patients at high risk of failure. In fact, a significant proportion of the patients included had one or more risk factors for extubation failure [2]. Interestingly, the effects of high-flow nasal cannula (HFNC) on respiratory effort were consistent among the study population, independent of their number of risk factors for extubation failure. Second, although the concern regarding a crossover design in unstable settings is theoretically correct, as there may be a period effect and patients may spontaneously exhibit systematic changes in the outcomes of interest from the first to the second period [3], our data showed that this phenomenon did not occur in our study. There was no physiologic change between the first and the second periods in any of the variables analyzed as shown in Table S2. Nor was there a carryover effect, other potential limitation of crossover designs which we ruled out. The crossover design is not novel in critical illness. It has been applied in several physiologic studies in acute respiratory failure [4–6], spontaneous breathing trials (SBT) [7], and the early postextubation phase [8–10]. All these studies have made relevant contributions to understand the physiologic effects of non-invasive respiratory support highlighting the value of a crossover design when taking care of the potential limitations. Therefore, we do not agree that the study lacked a comprehensive approach. Moreover, one of its major strengths is the extensive assessment of several variables related to the different mechanisms of weaning failure, which is the defining characteristic of a comprehensive physiologic study.

Toumi et al. also proposed that collecting more frequent data within each 1-h treatment period would have provided additional relevant information (e.g. every 10 min). We do not agree with this proposition because transient changes which are not sustained toward the end of the study period may be of limited interest. They may be explained by an episode of cough or a patient movement. The data presented were obtained from the last 5 min of each period. Special care was dedicated so that the time frame analyzed was not affected by transient changes due to movements, cough, or artifacts, but instead that it reflected the effective respiratory status of the patient toward the end of each study period. If the patient consistently improved or deteriorated within each 1-h treatment period, this was clearly reflected in the single assessment presented. Regarding the inclusion of physiologic data from the end of the spontaneous breathing trial, we agree that it would have been a valuable contribution, and we plan to include this assessment in futures studies. The inclusion of passive mechanics, although potentially of interest, in this clinical setting is complex as patients are already in assisted or spontaneous modes and awake at the time of inclusion.

Finally, Toumi et al. indicate that our study failed to find a minimal clinically important difference. This concept applies more to patient centered outcomes than to a physiologic study. However, our study demonstrated that compared to standard oxygen, HFNC decreased respiratory effort as manifested by a 46% mean reduction in pressure time product per minute. This is clearly a physiologically important difference which probably explains the important clinical differences found in clinical trials [11], and the strong recommendation to prefer HFNC over standard oxygen to prevent extubation failure [12–15].

## Data Availability

My manuscript has no associated data.

## Abbreviations

HFNC: High-flow nasal cannula
NIV: Non-invasive ventilation
SBT: Spontaneous breathing trial
